# Analysis of Human Exercise Health Monitoring Data of Smart Bracelet Based on Machine Learning

**DOI:** 10.1155/2022/7971904

**Published:** 2022-06-08

**Authors:** Xiaoge Ma

**Affiliations:** School of Sports and Leisure, Guangdong Ocean University, Zhanjiang, Guangdong,524088, China

## Abstract

The smart bracelet has become a hot-selling commodity, according to a daily consumption survey. Based on people's interest and concern for their health, the smart bracelet, as a design and application for achieving healthy weight loss monitoring, is quickly becoming a popular new favorite. This bracelet detects fat using the near-infrared diffuse reflection principle, with the goal of assisting people in controlling and maintaining a healthy weight. A large amount of data has been accumulated in all walks of life due to the development of the Internet network and data storage technology. As a result, the emergence of machine learning plays a critical role in the data analysis of human sports health monitoring of smart bracelets. Based on machine learning, this paper investigates the data analysis of human sports health monitoring smart bracelets. When the population index reaches 50 in the analysis of health monitoring data, the average accuracy of data mining is 86.8 percent, the average accuracy of the association rule algorithm is 85.9 percent, the average accuracy of the collaborative filtering algorithm is 84.3 percent, and the average accuracy of the machine learning algorithm is 90.1 percent in this paper. Among the four algorithms, the method presented in this paper is clearly the most effective, stable, and accurate. The system's stability and accuracy have been greatly improved by the addition of GPS-assisted and hand-up misjudgment algorithms. Because the smart bracelet is inexpensive, easy to wear, and consistent with consumer psychology, it is becoming increasingly popular to use it to monitor the human body's sports health.

## 1. Introduction

With the increasingly fierce social competition, young people's work pressure is gradually increasing, and they have little time to pay attention to the travel activities and vital signs of the elderly and children, such as obesity caused by less exercise, falling alarm of the elderly, exercise monitoring and calorie consumption, and measurement of heart rate and exercise [1, 2]. The smart bracelet has become a hot-selling commodity, according to a daily consumption survey. Based on people's current interest and focus on health, the smart bracelet, as a design and application for achieving healthy weight loss monitoring, is quickly becoming a popular new favorite. This bracelet detects fat using the near-infrared diffuse reflection principle, with the goal of assisting people in controlling and maintaining a healthy weight [3]. Currently, scientific researchers in the field of sports health use the traditional acceleration motion sensor worn at the waist to monitor physical activity, obtain effective data for analysis and processing, provide a scientific foundation for mass sports fitness, and increase the scientificity of mass sports fitness. However, the bracelet must use its own algorithm to calculate the acceleration vector length changes in three directions of wrist movement during human movement. However, because the wrist's movement direction cannot be determined, calculating the number of steps of the bracelet in software requires high accuracy. Because the traditional acceleration motion sensor is small and convenient and does not require subject review, it can effectively reduce recall bias and accurately provide information about energy consumption, frequency, intensity, and duration of physical activities through the validity tests of various methods, and it has been widely accepted and applied in scientific research.

With the development of the Internet network and data storage technology, a large amount of data has been accumulated in all walks of life. The application of health monitoring data mainly refers to the use of Internet technology and big data technology [4, 5] for data mining and analysis, the analysis and integration of health information and data at all levels, and the improvement of health services, to make the operation of various medical industries at all levels more efficient and make the services of various medical industries at all levels better under the background of China's informatization. Machine learning [6–8] can not only be applied to the preprocessing stage of monitoring data but also, more importantly, it is necessary to establish a learning model and actively learn the information contained in the data to evaluate the safety of the structural state and reflect the health state in time. In the face of a large amount of information generated all the time, the traditional manual method is simply unable to make efficient and rapid judgment [9, 10]. Machine learning first appeared in the last century. Learning in machine learning actually wants to express learning from data. It consists of three parts: unsupervised learning, semisupervised learning, and supervised learning. Machine learning uses computers to simulate human learning activities. Today, machine learning is the main way to deal with big data. As a multidisciplinary field, machine learning is widely used [11].

The use of machine learning in the analysis of human exercise health monitoring data from a smart bracelet is reflected not only in the preprocessing of raw data and the efficient operation of the learning model but also in the “iterative optimization” of the machine learning model. The most significant difference between machine learning and traditional manual quantitative and qualitative analysis is that the algorithm has the property of “iterative optimization,” which means that the learning model established by machine learning can be optimized step by step. The more data samples there are, the more effective the information the learning model has, and the model's accuracy and precision can be continuously improved [12, 13]. To accurately and efficiently synthesize existing motion capture data into a new motion sequence, three basic problems must be solved: how to establish a mathematical model of human motion, how to reduce the dimension of the motion data, and how to quickly and accurately synthesize a new motion sequence [14]. Because these three fundamental problems are so closely related, the applicable dimensionality reduction method and synthesis algorithm are determined by the specific mathematical model. Simultaneously, the specific synthesis algorithm and dimensionality reduction method require the assistance of specific mathematical models. The accelerometer principle is also used by the smart bracelet to monitor physical activity. By connecting with the corresponding mobile phone application software, the public can intuitively obtain physical activity index data such as energy consumption and daily steps, thus effectively monitoring the realization of long-term or short-term physical activity goals. In addition, because the smart bracelet is affordable, easy to wear, and in line with the public's consumption psychology, it is becoming more and more popular to apply the smart bracelet to monitor the level of human sports health [3]. The innovations of this paper are as follows:

1. Based on machine learning, this paper builds an intelligent Bracelet human motion health monitoring data model. The machine learning process entails training the model on a large number of task-related data sets; iterating the training model continuously to obtain a reasonable model fitting the data set; applying the training adjusted model to the real scene. The selection of input parameters, output parameters, and learning algorithm is part of the process of developing a predictive GRF learning model.
2. A data collection system for intelligent Bracelet human movement health monitoring is built. Although researchers are dedicated to testing the validity of sports intelligent Bracelet monitoring physical activities, the intelligent Bracelet human sports health monitoring data system is still in its early stages. This paper analyzes and summarizes existing research results and research progress by combing the relevant literature on the validity of sports intelligent Bracelet evaluation steps, energy consumption, distance, and physical activity intensity, in order to serve as a reference for future research.

The following sections comprise the overall structure of this paper: The first chapter discusses the history and significance of human movement in intelligent bracelets before moving on to the main work of this paper. The second chapter focuses primarily on the related work of intelligent Bracelet human movement both at home and abroad. The algorithm and model of machine learning are introduced in the third chapter. The fourth chapter describes the implementation of human motion health monitoring data from an intelligent bracelet, as well as the experimental part's analysis. The fifth chapter is a synopsis of the entire document.

## 2. Related Work

### 2.1. Research Status at Home and Abroad

Ciocca Gianmarco proposed that the health monitoring system of human movement of the intelligent bracelet is indeed much more efficient than the traditional manual method, but it also brings many challenges while providing real-time data: How to transform the noisy original data into processable and effective information, and how to further improve the computing power of the algorithm model to process the increasing real-time monitoring data [15]. Tang et al. proposed that while adding some new functions, the design and development of an intelligent bracelet will take the advantages and disadvantages of the 37-degree bracelet as a reference. On the basis of avoiding similar disadvantages, some design improvements have been added to the bracelet developed this time [16]. Garofolini a et al. proposed that human motion recognition technology based on acceleration sensors has been a hot research topic in the field of human motion recognition and health monitoring data analysis for a long time. It aims to use various information or data processing technologies to establish a behavior perception platform for the purpose of finding the representation, modeling, and prediction methods of health monitoring data movement [17]. GFA B et al. proposed that the core of the health monitoring system of human movement of the intelligent bracelet is a set of systems that analyzes the output response state based on structural information, historical records, and real-time data collected by sensors to evaluate the use and safety [18]. Vaz *d* v et al. proposed that in recent years, artificial intelligence technology is in the stage of rapid development, and the big data analysis industry is booming. Borrowing artificial intelligence technology is not only the trend of human motion recognition but also the inevitable result of the development of the motion recognition field [19]. Aerenhouts Dirkdirk proposed that there are two kinds of posture recognition technologies: wearable and nonwearable. As the name implies, wearable technology refers to the human body posture recognition technology, such as image recognition, in which the posture recognition device is not in direct contact with the human body. Compared with nonwearable, wearable human posture recognition technology has the advantages of unlimited space and has a better development space in research and application [20]. Julie M et al. proposed that for common physical activities, using the acceleration and gyroscope in smartphones to recognize human motion can achieve good accuracy. It has great potential to develop timely action classification and recognition and feedback programs with intelligent mobile terminals [21]. Higueras-Fresnillo S et al. proposed a PHM system based on Bayesian estimation theory and general particle swarm filtering framework, which integrated the modules of human motion data processing, eigenvalue extraction, fault diagnosis, and failure prognosis of the smart bracelet. Purdue University's Intelligent Process System Laboratory has put forward different solutions based on knowledge discovery, neural networks, and statistical analysis in the field of risk identification of complex systems [22]. Yanci Javier proposed to use the smart bracelet to recognize human motion, which is basically the processing of the information of human motion captured by sensors in the bracelet. Considering that the research object of this subject is people, which have wide differences and the quality of smart bracelets worn is also different, the acceleration sensor equipped with each smart bracelet is used as the human motion data acquisition module based on human motion recognition of smart bracelets [23]. Monajati Alireza Larumbe proposed that the basic human motion recognition method of a smart bracelet is the threshold detection method. That is, by judging whether the sensor value exceeds the set value, several kinds of actions can be distinguished. For example, several ongoing basketball playing actions can be identified by the data interval and vibration amplitude collected by the acceleration sensor [24].

### 2.2. Research Status of Human Motion Health Monitoring Data of Intelligent Bracelet Based on Machine Learning

This paper uses machine learning to analyze the human movement health monitoring data of an intelligent Bracelet. In comparison to previous studies, this method has the functions of step counting, positioning, and vital sign monitoring. It can calculate the number of steps a person walks using the pedometer function and the movement distance using the GPS positioning function to calculate the number of calories consumed, making it easy for people to understand their own and family members' movements. A heart rate sensor is a sensor that detects the rate of the heart. Accurate motion analysis can be achieved by collaborating with other sensors. Typically, three methods are used: photoelectric transmission measurement, test ECG signal measurement, and vibration measurement. When the heart is bleeding, the blood absorbs light of a specific wavelength. This characteristic of blood is used by the photoelectric transmission measurement method to determine heart rate. In light of this phenomenon, this paper performs hardware circuit design and software debugging, relying on the intelligent bracelet as the medium, so that the bracelet can not only realize indoor high-precision positioning but also upload the carrier's vital signs information to the positioning server, so that family members can accurately understand the internal position and physical condition in time. Furthermore, people may feel uneasy in the continuous monitoring environment for an extended period of time. This uneasy state will impair a person's activity performance and lead to inaccurate behavior detection. Furthermore, the video acquisition results will contain information about the life of the monitoring object, and improper use will result in the disclosure of users' personal information. Researchers have increasingly introduced various methods in machine learning into the research of human motion health monitoring of intelligent bracelets due to the high-dimensional characteristics and great temporal and spatial correlation of human motion data, which has made great progress in the research of human motion health monitoring of intelligent bracelets.

## 3. Algorithm and Model of Machine Learning

**Figure 1:**
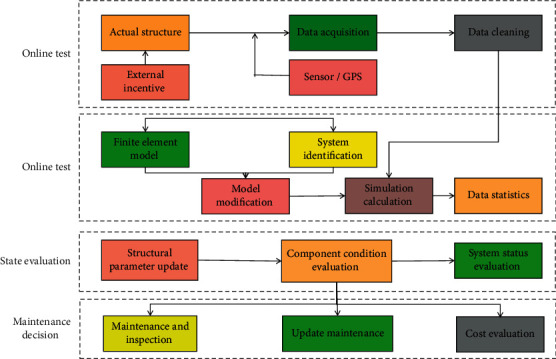
Machine learning health monitoring model.

Simulated learning is another name for machine learning. In layman's terms, this means that computers are learning how to think like humans. It stimulates the human learning process in order to achieve learning. Computers are used to simulate human learning activities in machine learning. Following the computer learning process, it will acquire data information and new knowledge in order to continuously improve its performance. Machine learning is defined as “a process in which a model learns and explores the existing connections and potential information among data based on the existing information set, in order to continuously optimize its own performance.” When compared to traditional computing methods, the most distinguishing feature of machine learning is its “self-learning and self-adaptation.” It has achieved great success in many fields such as finance and biology by fully drawing on the working mechanism of the human brain “neurons are connected hierarchically and information is updated iteratively.” The decision tree algorithm is a popular machine learning algorithm that is used not only for classification but also for regression. This algorithm serves as the foundation for many other advanced algorithms. Machine learning is classified into two types: supervised learning and unsupervised learning, depending on whether the training samples are labeled manually in advance. Supervised learning is a learning method that uses labeled data as the training data set, learns the rules for dividing objects from the training data set's characteristics, applies this rule to predict classification results on the test data set, and outputs the labeled results. Unsupervised learning is a learning method that is used to deal with data that has not been labeled or classified. Its goal is to discover potential relationships and statistical laws between data in order to determine the characteristics of the sample data. A decision tree is an inverted tree structure composed of a root node, an inner node, a leaf node, and an edge. The root node is the highest node, the leaf node provides the judgment conclusion, and the internal node is a relative concept. The purpose of building a decision tree is to perform classification or regression. The purpose of this paper is to investigate classification or the relationship between target variable categories and attributes. We can predict the category of the test data set once the relationship is built. At the moment, more research is needed on the high integration of “data collection, data preprocessing, data analysis, model evaluation, information visualization, and early warning mechanism.” Figure 1 depicts the basic structure of a common structural health monitoring system, which is made up of five major components: “online test, real-time analysis, damage identification, condition assessment, and maintenance decision.”

The main information about each meal in the file has two parts. One is the position and rotation information of the root joint. The second is the rotation information of each joint. Therefore, each frame of human motion can be expressed as follows:

(1) $$\begin{array}{c} f_{i}=\left(p_{i},r_{i}^{1},r_{i}^{2},\ldots r_{i}^{n}\right), \end{array}$$

where *f*_*i*_ represents the data of the *i*, *p*_*i*_ represents the position and rotation information of the root joint in the frame, *r*_*i*_^1^, *r*_*i*_^2^,…*r*_*i*_^*n*^ represents the rotation angle *r* ∈ *R*^3^ of each joint, and *n* is the number of joints defined in the *ASF* file.

Therefore, a motion sequence defined by the *ASF* − *AMC* capture data file can be expressed as follows:

(2) $$\begin{array}{c} \text{motion}=\left(f_{1}^{T},f_{2}^{T},\ldots f_{m}^{T}\right), \end{array}$$

where *f*_*i*_^*T*^ is the transpose of *i* frame data *f*_*i*_, and *m* is the number of rounds of the motion sequence.

Mathematicians have proved that the connection of a rotation sequence is equivalent to a single rotation. Therefore, any angular displacement can be expressed as a single rotation of an object around a certain axis. That is, a corner of the axis is used to describe (*n*, *θ*)*s* orientation, *n* represents the unit vector of the axis, and *θ* represents the angle of rotation around the axis. This method is seldom used in implementation, usually replaced by Euler angle or quaternion.

(3) $$\begin{array}{c} q=\left[\text{cos}\frac{\theta }{2},n\text{sin}\frac{\theta }{2}\right],\\ q=\left[\text{cos}\frac{\theta }{2},\left(n_{x}\text{sin}\frac{\theta }{2}\right)n_{y}\text{sin}\frac{\theta }{2}n_{x}\text{sin}\frac{\theta }{2}\right]. \end{array}$$

Therefore, if the quaternion *p*=[0, (*x*, *y*, *z*)] is used to represent a standard point in the three-dimensional space, and the corner of the rotating axis is (*n*, *θ*), let the quaternion *q*=[cos*θ*/2, *n*sin*θ*/2] , and the formula can show that the point *p* rotates by *θ* degrees around the *n* axis.

(4) $$\begin{array}{c} p^{\prime }=qpq^{-1}. \end{array}$$

According to the connectivity of rotation, if the point *p* rotates through quaternions *a* , *b* , according to the associative law of quaternions, it can be expressed as follows:

(5) $$\begin{array}{c} p^{\prime }=b\left(apa^{-1}\right)b^{-1}\\ =\left(ba\right)p\left(a^{-1}b^{-1}\right). \end{array}$$

Therefore, the rotation of a certain point in three-dimensional space can be expressed by the connection of multiple quaternions.

Let two similar motion sequences *T*, *R*, whose sequence lengths are *t*, *r*, respectively. Normally, *t* ≠ *r* can be known from the following formula:

(6) $$\begin{array}{c} T=\left[T_{1}\;T_{2}\ldots T_{t}\right],\\ R=\left[R_{i}\;R_{2}\ldots R_{r}\right]. \end{array}$$

Among them, the dimension of *T*_*i*_, *R*_*j*_(*i* ∈ [1, *t*], *j* ∈ [1, *r*]) is *P* . The purpose of *DT*  *W* algorithm is to find an optimal synchronization path by pattern matching the two motion sequences, so that the two motion sequences have the same length and retain the most complete data information, that is, to find the sequence *F*^*∗*^ in a grid

(7) $$\begin{array}{c} F^{*}=\left\{c\left(1\right),c\left(2\right),\ldots ,c\left(k\right),\ldots ,c\left(K\right)\right\},\text{max}\left(t,r\right), \end{array}$$

where *c*(*k*) can be expressed as follows:

(8) $$\begin{array}{c} c\left(k\right)=\left[i\left(k\right),j\left(k\right)\right]. \end{array}$$

*c*(*k*) means that when the *k* frame is matched, comparing the *i*(*k*) frame of sequence *T* with the *j*(*k*) frame of sequence *R* , *c*(*k*) can be regarded as a point in *t* × *r* grid. With the increase of *k* , it moves in *t* × *r* grid, and the curve formed by its moving track is the matching path. Therefore, *F*^*∗*^ is also called time bending function.

Since the generated shortest matching path must be equal to the length of motion sequence *T* , *k*=*t* and *k*=*i* can be obtained

(9) $$\begin{array}{c} F^{*}=\left\{c\left(1\right),c\left(2\right),\ldots c\left(i\right),\ldots ,c\left(t\right)\right\}. \end{array}$$

Obtain the shortest matching path *F*^*∗*^ , an asymmetric algorithm called *DT*  *W* . Including all frames of the standard reference sequence *T* , and some posts in the sequence *R* to be aligned are compressed or stretched due to different local features from the standard reference sequence. After this processing, the motion data before and after alignment are different, resulting in the following effects.

**Figure 2:**
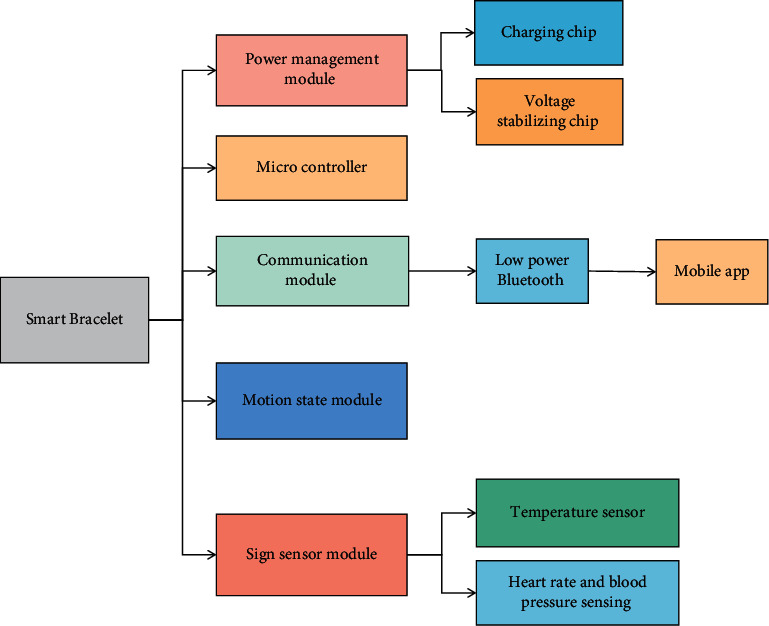
Functional module diagram of intelligent Bracelet system.

In the algorithm of machine learning, the artificial neural network is a mathematical model which can imitate brain neurons to a certain extent. In the algorithm of machine learning, artificial neural network processes data in this way, and the application of an artificial neural network is also very much. The process of machine learning includes using a large number of task-related data sets to train models. Through the error of the model on the data set, the model is repeatedly trained, and a model that fits the data set reasonably is obtained. Apply the trained and adjusted model to the real scene. A process of building a predictive GRF learning model includes the selection of input parameters, output parameters, and learning algorithm. Decision tree algorithm has the general characteristics of machine learning algorithms, which build models based on specific data, so it is different from traditional statistical models. It does not need prior assumptions, and it does not need data to obey any probability distribution. This is actually a very good feature because the actual data is generally difficult to meet the settings of those boxes. The core idea of deep learning is that unsupervised learning from bottom to top trains the data layer by layer and extracts the statistical characteristics nonlinearly, constantly updates and establishes the mapping relationship in the calculation, establishes the initialized network, and iteratively updates and optimizes the network parameters by using supervised learning from top to bottom, thus achieving the expected effect. Different from the shallow network structure, the multihidden layer structure of deep learning can break through the parity problem of the shallow structure, can complete the approximation of complex functions, realize the accurate processing of complex problems, and has good generalization ability. The smart bracelet system designed in this paper consists of two parts: smart bracelet and ultrawideband positioning system. The smart bracelet can be divided into six modules according to its functions, and the relationship between the components of the modules and the sensors is shown in Figure 2.

The power management module, which consists of a charging chip and a voltage stabilizing chip, is one of the six modules of the smart bracelet: A microcontroller can control data acquisition and processing; There is a mobile node and low-power Bluetooth communication module; Gyroscopes are used to realize the motion state module of step counting and motion state analysis. TFT-LCD display screen is used in the display module. A sign sensor module includes a heart rate and blood pressure sensor, a blood oxygen sensor, and a temperature sensor. The sensor's data can be transmitted to the mobile app via low-power Bluetooth, and it can also be viewed on the display screen. Second, the mobile node in the UWB positioning system can communicate with the basic positioning wireless network to master the accurate location of the bracelet carrier in real time. Traditional signal processing and kinematics methods have lost their advantages in these technologies due to the rise of machine learning methods because traditional methods cannot well model some complex characteristics of the above motion data, whereas appropriate machine learning methods can not only model them but also effectively use these characteristics to complete specific tasks. Although the tasks of motion restoration and motion denoising differ slightly, the former is dedicated to regaining the lost joint position during the capture process, while the latter is dedicated to combating noise, but their methods are similar. As a result, they are not deliberately distinguished when introducing the current state of motion restoration research.

## 4. Realization of Monitoring Data of Human Exercise Health with Intelligent Bracelet

### 4.1. Design of Intelligent Bracelet Human Exercise Health Monitoring Data System

The monitoring data of human exercise health of intelligent bracelets is the key technology and the most difficult link in the process of exercise data reuse. The analysis of human exercise health monitoring data system under machine learning has high dimensions, a large amount of information, and a complex structure, all of which bring challenges to exercise synthesis. The synthesized motion data will eventually be used for viewing, and the human eye is very good at finding out the incongruities in the motion, which puts forward higher requirements for motion synthesis. Usually, there are many physical quantities in the data of human motion recognition, and different physical quantities have different measurement methods, so they cannot be directly compared with each other. Therefore, it is necessary to normalize the feature vectors so that different feature vectors are in an equal and comparable position. In this paper, the normalization function map min-max in Matlab is used to normalize the characteristic data. After normalization, the restriction of units between different eigenvalues is eliminated, and the differences between different individuals are also reduced, and they are converted into pure values to facilitate feature extraction and comparative analysis. In the data system of human exercise health monitoring with an intelligent bracelet based on machine learning, users can switch measurement functions and observe data through touch screen, so three interfaces are designed according to bracelet functions. One is a clock interface to display real-time time. The main menu interface allows users to independently select the functions to be measured. The other is the display interface to display the measured data in real time. The structure of the sports bracelet includes a system processor module, sensor module, GPS module, and OLED display module. MK60DN512ZVLQ10 chip is adopted as the processor, which is a 32-bit processor with ARM-CORTEX-M4 as the core introduced by NXP. Whether it is used for scientific research or mass fitness, it is very important to evaluate its validity.

At present, under the intelligent Bracelet human sports health monitoring data system, although researchers are committed to testing the validity of sports intelligent Bracelet monitoring physical activities, it is still in its infancy. This paper analyzes and summarizes the existing research results and research progress by combing the relevant literature on the validity of sports intelligent Bracelet evaluation steps, energy consumption, distance, and physical activity intensity, so as to provide reference for further research. The sensor module includes an acceleration sensor mpu6050, which includes a multichannel ad, which can convert the obtained analog data into digital signals and transmit them to the processor through IIC. Through processing, it can obtain the human motion state, realize the step measurement function and control the OLED on and off function. The pulse heart rate sensor pulse sensor outputs analog signals, which are processed by ad to detect human heart rate data in real time. The GPS module sends data to the processor in the form of serial port to obtain position coordinate information. Therefore, motion synthesis has always been a very hot research topic. With the deepening of research, some excellent motion synthesis algorithms have emerged. Among them, the data-driven motion synthesis method can maintain the temporal and spatial characteristics of motion data, so it has been widely studied and applied. According to its different implementation methods, it can be divided into four categories: motion mixing, motion synthesis based on graph search and motion transition, parametric motion synthesis, and motion synthesis based on deep learning. Then machine learning is used to analyze the acceleration data of the smart bracelet to realize the attitude recognition on the mobile device. Finally, the comparative analysis of human motion based on various methods verifies that the analysis system of human motion health monitoring data system of intelligent bracelet based on machine learning is more accurate than traditional recognition methods such as support vector machine, more convenient and more practical than computer-based analysis platform.

### 4.2. Experimental Results and Analysis

The type of SVM selected in this study is C-SVC. When studying the effect of the RBF kernel, the loss function C of C-SVC and the gamma function *G* of the RBF kernel have a major impact on the recognition accuracy. The labeled test set is brought into the classifier for verification, and the classification accuracy rate is 94.4652%. The results expressed in are shown in Table 1. The training time is 45.702 seconds, and the testing time is 0.0242 seconds.

As the gamma function does not need to be set for the linear kernel, this study only needs to set the loss function C of C-SVC when testing with linear kernel and bring the labeled test set into the classifier for verification, and the classification accuracy rate is 93.0495%. The results are shown in Table 2. The training time is 60.885 seconds, and the testing time is 0.023 seconds.

From Tables 1 and 2, it can be seen that both RBF kernel SVM algorithm and linear kernel algorithm can achieve more than 90% recognition rate for the problems studied in this paper, and the recognition accuracy of RBF kernel SVM classification algorithm is slightly higher than that of linear kernel SVM algorithm, and the memory occupation and training time of the former are better than those of the latter. It can be seen that the RBF kernel is more suitable for the human motion recognition algorithm studied in this paper.

**Figure 3:**
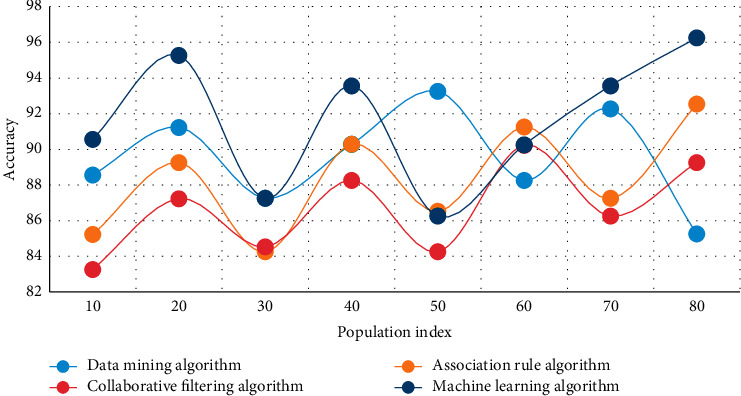
Comparison of several algorithms in health monitoring data analysis.

**Figure 4:**
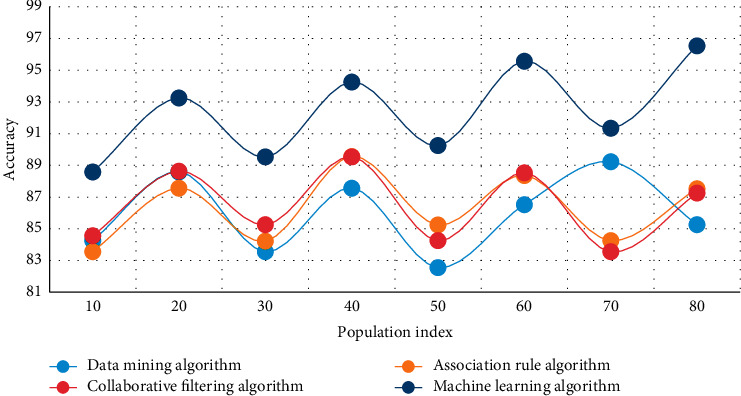
Comparison of several algorithms in health monitoring data analysis.

**Figure 5:**
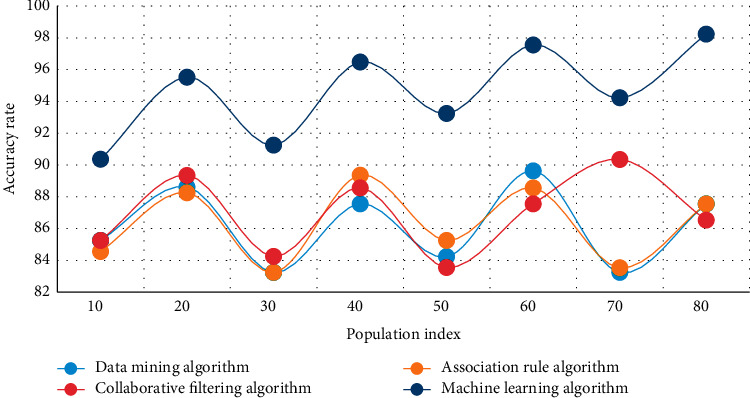
Comparison of several algorithms in health monitoring data analysis.

In this experiment, the human movement health monitoring data of intelligent bracelets are analyzed, and the data mining algorithm, association rule algorithm, collaborative filtering algorithm, and machine learning algorithm are used to compare the steps of left and right hands wearing bracelet randomly and test walking and running, respectively. The experimental results are shown in Figures 3–5.

**Figure 6:**
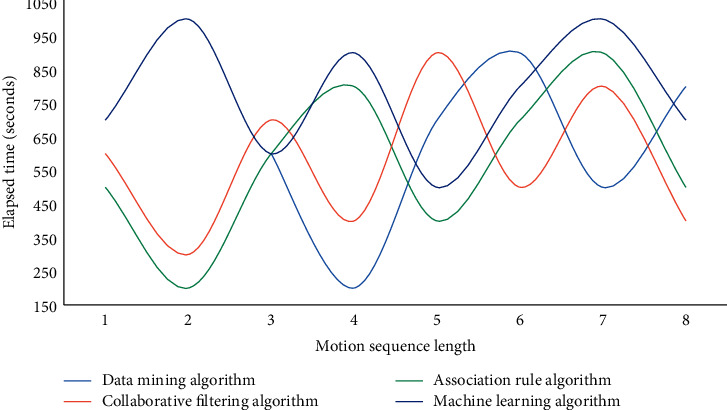
Time curves consumed by different algorithms on different length motion sequences.

**Figure 7:**
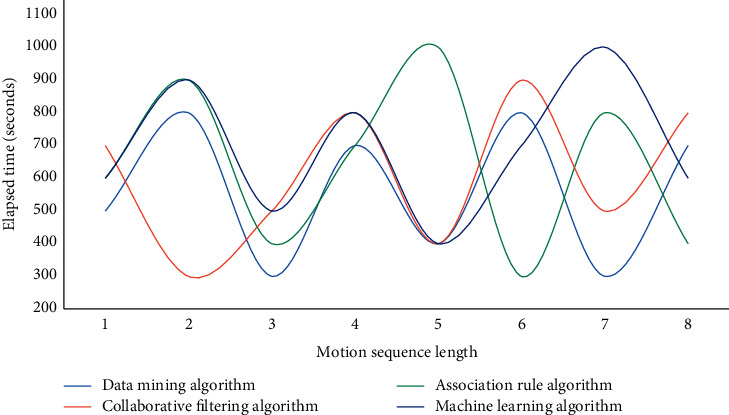
Time curves consumed by different algorithms on motion sequences of different lengths.

Figures 3–5 show that when the population index reaches 50 in the analysis of health monitoring data, the average accuracy of data mining is 86.8 percent, the average accuracy of the association rule algorithm is 85.9 percent, the average accuracy of the collaborative filtering algorithm is 84.3 percent, and the average accuracy of the machine learning algorithm is 90.1 percent. Among the four high-accuracy algorithms, this method is clearly the most effective and stable. The addition of GPS assistance and a hand raising misjudgment algorithm improves the system's stability and accuracy significantly. The monitoring data of human exercise health of smart bracelets are analyzed in this experiment, and the curves of time consumption increasing with the sequence length of the data mining algorithm, association rule algorithm, collaborative filtering algorithm, and machine learning algorithm are used in this paper, respectively. The experimental results are shown in Figures 6 and 7.

As can be seen from Figures 6–7, the time consumption curve of the machine learning algorithm in this paper is the highest among the four algorithms, and the linear complexity of the algorithm is verified from an experimental point of view. The time consumption curves of the data mining algorithm, association rule algorithm, and collaborative filtering algorithm indicate that they have quadratic complexity, and the linear complexity is another advantage of the algorithm in this chapter. Because accelerometer data and gyroscope data are three-dimensional data, and there are many sampled data, the extracted feature dimensions are too large, which is not conducive to the subsequent classification and recognition. At the same time, more redundant features will also reduce the classification accuracy so that the most effective features can be selected to achieve the effect of dimension reduction and improve the classification accuracy.

## 5. Conclusions

In this paper, a set of human motion recognition systems is developed using the intelligent bracelet's built-in acceleration sensor. In this paper, machine learning is used as a human motion recognition algorithm, and for the first time, this method is realized by an intelligent bracelet on a mobile terminal. This method is then contrasted with the data mining algorithm, the association rule algorithm, and the collaborative filtering algorithm. When the population index reaches 50 in the analysis of health monitoring data, the average accuracy of data mining is 86.8 percent, the average accuracy of the association rule algorithm is 85.9 percent, the average accuracy of the collaborative filtering algorithm is 84.3 percent, and the average accuracy of the machine learning algorithm in this paper is 90.1 percent. This method is clearly the most effective and stable of the four high-accuracy algorithms. The addition of GPS assistance and a hand raising misjudgment algorithm greatly improves the system's stability and accuracy. With the rapid advancement of science and technology in recent years, we believe that the development of smart bracelets will be extremely beneficial. The combination of smart bracelets with sports, medical treatment, and mobile payment will make our lives more convenient and fast in the future. Current machine learning models, on the other hand, are based on smaller data sets. Data sharing can provide larger data sets for developing a comprehensive learning model for a broader range of people. Simultaneously, the current learning model is primarily used for walking, running, and a few special movements. Its wide range of applications in various human movements warrants further investigation.

## Figures and Tables

**Table 1:** Forecast details of RBF core test set.

	Walk	Go upstairs	Sit-up	Run	Go downstairs	Wipe	%
Walk	102	6	1	2	1	4	85.82
Go upstairs	3	61	1	0	3	1	88.56
Sit-up	2	0	82	0	0	0	98.82
Run	0	1	0	114	1	0	102
Go downstairs	6	4	0	0	104	2	89.73
Wipe	0	2	1	1	0	1	98.58

**Table 2:** Prediction details of linear kernel SVM test set.

	Walk	Go upstairs	Sit-up	Run	Go downstairs	Wipe	%
Walk	96	12	1	2	3	4	70.28
Go upstairs	8	55	0	1	4	1	80.03
Sit-up	2	0	83	1	1	0	101
Run	1	1	1	112	0	1	102
Go downstairs	5	2	0	0	107	0	92.33
Wipe	3	2	1	1	0	131	102

## Data Availability

The data used to support the findings of this study are available from the corresponding author upon request.
